# Specific binding sites on Rhesus rotavirus capsid protein dictate the method of endocytosis inducing the murine model of biliary atresia

**DOI:** 10.1152/ajpgi.00308.2023

**Published:** 2024-06-11

**Authors:** Haley Temple, Bryan Donnelly, Sujit K. Mohanty, Sarah Mowery, Holly M. Poling, Rajamouli Pasula, Stephen Hartman, Akaljot Singh, Reena Mourya, Alexander Bondoc, Jaroslaw Meller, Anil G. Jegga, Kei Oyama, Monica McNeal, Paul Spearman, Greg Tiao

**Affiliations:** ^1^Department of Pediatric and Thoracic Surgery, Cincinnati Children’s Hospital Medical Center, Cincinnati, Ohio, United States; ^2^Southeast Poultry Research Laboratory, United States National Poultry Research Center, United States Department of Agriculture, Athens, Georgia, United States; ^3^Division of Gastroenterology, Hepatology and Nutrition, Cincinnati Children’s Hospital Medical Center, Cincinnati, Ohio, United States; ^4^Department of Environmental and Public Health Sciences, University of Cincinnati, Cincinnati, Ohio, United States; ^5^Division of Biomedical Informatics, Cincinnati Children’s Hospital Medical Center, Cincinnati, Ohio, United States; ^6^Division of Infectious Diseases, Cincinnati Children’s Hospital Medical Center, Cincinnati, Ohio, United States; ^7^Department of Pediatrics, University of Cincinnati College of Medicine, Cincinnati, Ohio, United States

**Keywords:** cholangiocyte, cytokine, endosome, Hsc70, TLR3

## Abstract

Biliary atresia (BA) is the leading indication for pediatric liver transplantation. Rhesus rotavirus (RRV)-induced murine BA develops an obstructive cholangiopathy that mirrors the human disease. We have previously demonstrated the “SRL” motif on RRV’s VP4 protein binds to heat shock cognate 70 protein (Hsc70) facilitating entry into cholangiocytes. In this study, we analyzed how binding to Hsc70 affects viral endocytosis, intracellular trafficking, and uniquely activates the signaling pathway that induces murine BA. Inhibition of clathrin- and dynamin-mediated endocytosis in cholangiocytes following infection demonstrated that blocking dynamin decreased the infectivity of RRV, whereas clathrin inhibition had no effect. Blocking early endosome trafficking resulted in decreased viral titers of RRV, whereas late endosome inhibition had no effect. After infection, *TLR3* expression and p-NF-κB levels increased in cholangiocytes, leading to increased release of CXCL9 and CXCL10. Infected mice knocked out for TLR3 had decreased levels of CXCL9 and CXCL10, resulting in reduced NK cell numbers. Human patients with BA experienced an increase in CXCL10 levels, suggesting this as a possible pathway leading to biliary obstruction. Viruses that use Hsc70 for cell entry exploit a clathrin-independent pathway and traffic to the early recycling endosome uniquely activating NF-κB through TLR3, leading to the release of CXCL9 and CXCL10 and inducing NK cell recruitment. These results define how the “SRL” peptide found on RRV’s VP4 protein modulates viral trafficking, inducing the host response leading to bile duct obstruction.

**NEW & NOTEWORTHY** In this study, we have determined that the presence of the “SRL” peptide on RRV alters its method of endocytosis and intracellular trafficking through viral binding to heat shock cognate 70 protein. This initiates an inflammatory pathway that stimulates the release of cytokines associated with biliary damage and obstruction.

## Graphical abstract

**Figure:**
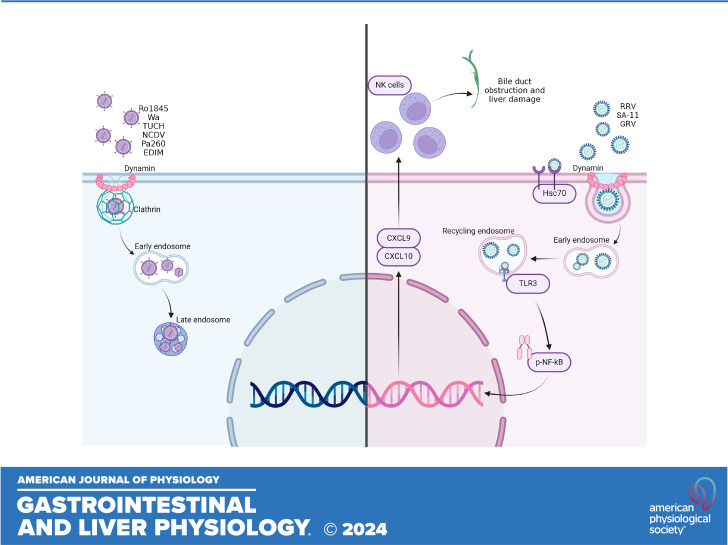

## INTRODUCTION

Biliary atresia (BA) is a devastating cholangiopathy of infancy that affects 1 in 15,000 births in the United States (1) and is the leading cause of pediatric liver transplantation (2). Without intervention via Kasai portoenterostomy (HPE) to restore bile flow, BA progresses to end-stage liver disease and ultimately death. Despite restoration of bile flow with HPE, 60% of patients still require liver transplantation before 20 yr of age (3). Although the etiology of BA remains elusive, the presence of various viruses, including rotavirus, reovirus, cytomegalovirus (CMV), human papillomavirus (HPV), and Epstein-Barr virus (EBV), in the explanted livers of patients with BA suggests a viral insult may initiate an immune response that progresses to BA (4). Additional evidence for viral induction of BA is the murine model of the disease in which newborn mice infected with Rhesus rotavirus (RRV) develop symptoms similar to human patients, including hyperbilirubinemia, jaundice, acholic stool, and growth retardation (5–7).

Rotaviruses are double-stranded RNA (dsRNA) viruses belonging to the *Reoviridae* family consisting of 11 segments of dsRNA encoding six nonstructural proteins (NSP1-6) and six structural proteins (VP1-4, VP6, and VP7) (8). The outer capsid is composed of proteins VP4 and VP7, which are involved in cellular binding and entry to susceptible host cells (9). Although RRV has the capacity to bind to various receptors on the cell surface, its VP4 protein contains the amino acid sequence “SRL” allowing it to specifically interact with heat shock cognate 70 (Hsc70), facilitating viral attachment and entry into cholangiocytes (10). The viruses found in human patients with BA listed above also contain the “SRL” peptide on a binding protein.

dsRNA viruses can use a multitude of entry pathways including clathrin-dependent and -independent, dynamin-mediated, and caveolin-mediated endocytosis, as well as pinocytosis. After cell entry, viruses progress to the early endosome and many progress to the late endosome (11). Viruses that do not progress further than the early endosome are labeled “early-penetrating,” whereas viruses that traffic to the late endosome are labeled “late-penetrating” with viral uncoating and membrane penetration being stimulated by the endosome’s acidic environment (11, 12). The method of endocytosis can be determined by a variety of factors, but the cell surface receptor used by the virus has been suggested to play a major role in establishing the viral entry and uncoating pathway (13, 14).

Hsc70 is a molecular chaperone involved in multiple intra- and extracellular activities and is expressed on the surface of multiple cell types (15). Intracellularly, Hsc70 participates in protein folding, degradation, and clathrin-mediated endocytosis (15–17). Previous studies have shown Hsc70 is upregulated during infection and inflammation (15, 18). When expressed on the cell surface, Hsc70 can be used by viruses in attachment and entry (19–22). We have shown that viral binding to Hsc70 through the “SRL” amino acid sequence contained in RRV’s VP4 protein is necessary to induce murine BA (10). Altering one amino acid in RRV’s “SRL” sequence to “SGL” (RRV^VP4-R446G^) renders it incapable of binding to Hsc70 and therefore unable to induce obstruction in the murine model of BA, conversely, Ro1845 is unable to bind to Hsc70 but when its “SGL” sequence is altered to “SRL” (Ro1845^VP4-G446R^), it can bind to Hsc70 and induce this obstruction (23, 24).

Expression of Toll-like receptor 3 (TLR3), a pathogen-associated molecular pattern receptor, is found in extrahepatic bile ducts of patients with BA (25). Activation of TLR3 occurs following stimulation with dsRNA, and it is found in abundance in the early endosome (26–28). TLR3 signaling activates NF-κB leading to proinflammatory cytokine release and recruitment of natural killer (NK) cells that are innate immune cells that target and kill infected cells (29, 30). Upon RRV infection, the ensuing cytokine release activates NK cells. Our previous study shows that RRV’s VP4 sequence-specific activation of NK cells is associated with cholangiopathy in murine BA by inducing cell death of both infected and noninfected cholangiocytes (31).

To understand how RRV can induce the murine model of BA, we analyzed the cell surface receptors used by other rotavirus strains, both those capable and incapable of inducing BA. We demonstrated that RRV’s VP4 “SRL” peptide binding to Hsc70 altered RRV’s method of endocytosis and intracellular trafficking, triggering the cholangiocyte response that governs biliary obstruction.

## EXPERIMENTAL PROCEDURES

### Cells, Viruses, and Animals

Mouse cholangiocyte cell line kindly provided by James Boyer (Yale Liver Care Center, Hartford, CT), MA104 cells (BioWhittaker, Walkersville, MD), HeLa wild-type (WT) (Ref. AB255928, Abcam, Waltham, MA), and HeLa cells knocked-out for Hsc70 (HeLa Hsc70 KO) cells (Ref. AB265664, Abcam) were cultured as previously described (32). Rotavirus strains used were RRV (Dr. Harry Greenberg, Stanford University, Palo Alto, CA); SA-11, EDIM, NCDV, and Wa (Monica McNeal, Cincinnati Children’s Hospital Medical Center); TUCH (Dr. Karol Sestak, Tulane National Primate Research Center and Tulane University School of Medicine); GRV (Dr. Osamu Nakagomi, Nagasaki University); PA260 (Dr. Vito Matella, University of Bari Aldo Moro); Ro1845 (Dr. Yasutaka Hoshino, National Institute of Allergy and Infectious Disease), and the two mutant strains: RRV^VP4-R446G^ (24) and Ro1845^VP4-G446R^ (33). All strains were propagated in MA104 cells as previously described (24, 33, 34).

WT-BALB/c mice (Ref. 047-US, Envigo Labs, Indianapolis, IN) and TLR3 KO on a BALB/c background [Ref. C.129S1(B6)-Tlr3tm1Flv/J, Oriental BioService, Ukyou-ku, Kyoto, Japan] were housed in micro isolator cages in a virus-free environment with access to sterilized chow and water ad libitum. All animal research was performed in accordance with regulations and protocols approved by the Institutional Animal Care and Use Committee at Cincinnati Children's Hospital Medical Center (Protocol No. IACUC2022-0048).

### Inhibition of Cell Infections

Cholangiocyte cells grown to confluency in 48-well plates were pretreated with increasing concentrations of sucrose (Ref. BP220-212, Thermo Fisher, Waltham, MA) or PITSTOP 1–25 (Ref. AB144651, Abcam) for 1 h (both clathrin pathway inhibitors), Dynasore (Ref. 501011186, Thermo Fisher) for 6 h, or Dyngo (Ref. AB120689, Abcam) for 30 min (both dynamin pathway inhibitors). After pretreatment, cells were washed twice with media, inoculated with virus at a multiplicity of infection (MOI) of 0.5, and incubated for 1 h at 37°C. Cells were washed twice, overlaid with media, and incubated at 37°C for 18 h. For experiments involving EGA [4-bromobenzaldehyde-*N*-(2,6-dimethylphenyl)semicarbazone, (*E*)-2-(4-bromobenzylidene)-*N*-(2,6-dimethylphenyl)hydrazinecarboxamide], a Late Endosome Trafficking Inhibitor (Ref. 5093060001, Sigma-Aldrich, St. Louis, MO), increasing concentrations were added to the overlay media. After infection, virus levels were assayed by focus forming assay (FFA).

### Quantification of Infectious Rotavirus

Infectious rotavirus was quantified by FFA as previously described (34, 35). Briefly, 96-well plates were seeded with MA104 cells and incubated for 4 days at 37°C. Once confluent, cells were washed, inoculated with serially diluted virus for 1 h, and incubated at 37°C. Cells were then washed with media, overlaid with DMEM containing 4 µg/mL of trypsin, and incubated for 14–16 h. After acetone fixation with cold 80% acetone for 15 min at –20°C, Guinea pig antirotavirus immunoglobulin G (IgG) antibody (1:1,000) (a generous gift from Monica McNeal) was added and incubated for 30 min. Wells were aspirated and washed with phosphate-buffered saline (PBS), and fluorescein isothiocyanate (FITC)-tagged goat anti-guinea pig IgG antibody (1:500) (Ref. 855387, MP Biomedicals, Solon, OH) was added for 30 min at 37°C. Wells were then washed twice and plates were scored using UV microscopy (×10 objective). Quantities of infectious virus were reported as focus-forming units (FFUs) per milliliter.

### Transfection of siRNA against Rab5 and Rab7

Transfection of siRNA to reduce Rab5 (Ref. L-040855-00-0005), Rab7 (Ref. L040859-02-0005), Rab11a (Ref. L-040863-01-0005), or TLR3 (Ref. L059850-00-0005) expression was carried out as previously described (32). The siRNA sequences (Supplemental Table S1) and nontargeting siRNA were purchased from Dharmacon (Lafayette, CO).

### Prediction of Protein-Protein Interaction

Experimentally resolved structures and AlphaFold models of Hsp110 orthologs and their complexes with Hsc70 were used to identify D390 site in Hsc70 as a putative binding site for RRV VP4 protein. First, the Hsp110 counterpart of RRV SRL (or extended VSRLY) motif with the central arginine (R) residue is identified based on sequence and local structure similarity as RVRPF in yeast and KVREF in human. With the use of the Hsp110:Hsc70 complex in yeast, this motif can be further mapped into the interaction interface with Hsc70, with the R390 residue in direct contact with D390 of Hsc70. X-ray-resolved structure of Hsp110:Hsc70 complex in yeast, PDB code 3C7N, was aligned with X-ray-resolved RRV VP4 structure PDB code 1SQL. The complete protein structure for the human Hsp110 protein has not been resolved experimentally, hence the use of AlphaFold software to model the human protein while using the complex of yeast homolog to map interaction sites.

### Viral Binding and Blocking with Synthetic Peptides

Synthetic peptides TRTRVSRLY, KVREF, DGEA, and GHRP were purchased from GenScript (GenScript, Piscataway, NJ) (10). Cholangiocytes and HeLa cells were grown to confluency in 24-well plates and treated as previously described (10). Briefly, media, cells, synthetic peptides, and inoculating virus were cooled to 4°C. Media was removed and treated with 1 mM of peptides diluted in DMEM for 1 h on a rocker at 4°C. Cells were washed twice, inoculated with an MOI = 0.5, and incubated for 1 h at 4°C. The inoculum was collected, along with media from two washes of the cells to remove the unbound virus. The cells then underwent two freeze-thaw cycles with the final cell fraction representing attached virus. The amount of bound and unbound virus was determined by FFA analysis and expressed as a percentage of attached virus versus the total amount of virus used to inoculate cells.

### Transduction of Mutant *HSPa8*

HeLa Hsc70 KO cells were seeded on 24-well plates and incubated for 24 h at 37°C. Wells that were 50% confluent were washed twice, overlaid with media, and transduced with a lentivirus (pLenti-C-Myc-DDK-P2A-Puro) containing a mutant human *HSPa8* plasmid, which encodes Hsc70, with amino acid aspartic acid 390 altered to an alanine generated by OriGene (Ref. CW307616, OriGene, Rockville, MD). Transduced cells were selected by 15 µg/mL treatment with puromycin and flow sorted for surface Hsc70 by Cincinnati Children’s Hospital’s Flow Cytometry Core.

### Detection of Proteins by Western Blot Analysis and Protein Array

Cell homogenates were diluted in 1x loading buffer, while supernatant was diluted in 5x loading buffer, separated on a 4–20% Tris-glycine gel (Invitrogen, Waltham, MA), transferred to polyvinylidene difluoride (PVDF) membranes (GE Healthcare, Pittsburg, PA), and blocked with 5% milk solution. Membranes were probed with a rabbit anti-Rab5 (1:1,000 dilution) (Ref. AB218624, Abcam), rabbit anti-Rab7 (1:1,000 dilution) (Ref. NBP267732, Novus Biologicals, Centennial, CO), rabbit anti-Rab11a (1:1,000 dilution) (Ref. 2413S, Cell Signaling, Boston, MA), rabbit anti-TLR3 (1:1,000 dilution) (Ref. AB137722, Abcam), rat anti-Hsc70 antibody (1:1,000 dilution) (Ref. 200062-710, VWR, Radnor, PA), mouse anti-DDK (1:1,000 dilution) (Ref. TA50011-30, OriGene, Rockville MD), rabbit anti-CXCL9 (1:1,000 dilution) (Ref. PI701117, Invitrogen), rabbit anti-CXCL10 (1:1,000 dilution) (Ref. 701255, Invitrogen), mouse anti-NF-κB (1:1,000 dilution) (Ref. 8242S, Cell Signaling), rabbit anti-phos-NF-κB (1:1,000 dilution) (Ref. 3039S, Cell Signaling), and mouse anti-actin antibody (1:5,000 dilution) (Ref. LMAB-C4, Seven Hills Bioreagents, Cincinnati, OH) as loading control. Blots were imaged using a Bio-Rad ChemiDoc MP(Bio-Rad Laboratories, Hercules, CA), and densitometry was quantified using ImageJ software (RRID:SCR_003070).

For protein arrays, 1 mL of supernatant from cholangiocytes infected with RRV or Ro1845 for 18 h was incubated with membranes provided in Proteome Profiler Mouse Chemokine Array (Cat. No. ARY020, R&D Systems, Minneapolis, MN). Membranes were processed according to the manufacturer’s protocol, imaged using a Bio-Rad ChemiDoc MP (RRID:SCR_014210, Software Image Lab version. 6.1.0 build 7, Bio-Rad Laboratories, Hercules, CA) and densitometry was quantified using ImageJ software (RRID:SCR_003070). Each pair of spots on the membranes are an antibody to a different chemokine.

### RNA Isolation and Real-Time PCR for *TLR3* Expression

Cholangiocytes were grown to confluency in 24-well plates and either exposed to serum-free media or infected with RRV, Ro1845, RRV^VP4-R446G^, or Ro1845^VP4-G446R^ for 8 h. Cells were lysed and the total RNA was extracted using the RNeasy Plus Mini Kits (Cat. No.: 74134, Qiagen, Germantown, MD) according to the manufacturer’s instructions. cDNA pools were generated using the High Capacity cDNA kit (Thermo Fisher), and mRNA expression for *TLR3* relative to glyceraldehyde-3-phosphate dehydrogenase was quantified by real-time PCR using SYBR Green on a Step One Plus Real-time PCR system (Applied Biosystems, Waltham, MA). The murine primers used for PCR were as follows: *TLR3*: sense, 5′- GATATCTCCCCTTCACCTTTCC-3′, antisense, 5′- CCTCCAGCAAGTCCTCATTTA-3′; glyceraladehyde-3-phosphate dehydrogenase (GAPDH): sense, 5′- CATTGACCTCAACTACATGGT-3′, antisense, 5′- ATGACAAGCTTCCCATTCT-3′.

### Viral Induction of Murine Model of Biliary Atresia

Cages of between 4 and 10 newborn pups were assigned to experiment groups using simple randomization with no sex bias. These mice were injected intraperitoneally within the first 24 h of life with 1.5 × 10^6^ FFU/pup of RRV, Ro1845, RRV^VP4-R446G^, Ro1845^VP4-G446R^, or saline as a control. Clinical symptoms of hepatobiliary injury (i.e., jaundice, acholic stools, and bilirubinuria) along with survival were assessed daily for 21 days. Subsets of pups were euthanized at 7- and 10-days postinfection, with their extrahepatic biliary tracts and livers harvested for histology or viral titers as determined by FFA.

### Laser Capture Microdissection on RRV-Infected Livers

Frozen tissue blocks were sectioned along the extrahepatic bile duct’s longitudinal axis at 7-µm intervals using a Microm HM 550 OVP cryostat (Thermo Fisher). Tissue sections were mounted on polyethylene naphthalate (PEN) membrane slides (MDS Analytical Technologies, Sunnyvale, CA) and stored at –80°C until use. Slides were quickly thawed, stained, and dehydrated according to the manufacturer’s instructions. Laser capture microdissection (LCM) was performed on biliary epithelial cells with the Veritas Microdissection System LCC 1704 (MDS) using the infrared capture laser. Typically, 5,000–7,000 cell sections were used and RNA was extracted from these cholangiocytes using the RNeasy Micro Kit (Qiagen, Valencia, CA) according to the manufacturer’s instruction and stored at –80°C.

RNA quality and quantity were assessed using the Agilent 2100 Bioanalyzer (RRID:SCR_019389) and RNA 6000 Pico Chip (Agilent Technologies, Palo Alto, CA).

### Target Amplification and Biotinylation

Amplification of sample RNA was required to generate necessary amounts of genetic material for microarray hybridization. This process was carried out using the WT Ovation Pico System (NuGEN, San Carlos, CA), which has been validated for amplification of picogram quantities of RNA for genome-wide studies (36). The amplified, complementary DNA (cDNA) product quality was again assessed by the Agilent 2100 Bioanalyzer and biotinylated using the FL-Ovation Biotin Module V2 (NuGEN).

### Microarray Analysis and Data Mining

Genomic material obtained from the extrahepatic bile duct of mice 7 days after injection with RRV or normal saline was hybridized to the GeneChip Mouse Exon 1.0 ST Array platform (Affymetrix, Santa Clara, CA), which contains 1.2 million probe sets per chip. Three samples representing both experimental groups were used. An initial genome-wide analysis was performed using GeneSpring GX (Agilent), identifying genes from RRV-challenged animals that were over or underexpressed 1.5-fold as compared with control samples using one-way analysis of variance (ANOVA) with post hoc *t* test. A false discovery rate (FDR) of 5% was used. Significance was set at *P* < 0.05. A subsequent, secondary analysis was performed using Ingenuity Pathway Analysis (RRID:SCR008653) (Ingenuity Systems, Redwood City, CA) (37). Dataset available through GEO accession number GSE267721.

### Histology

Ten-day postinfected liver and extrahepatic bile duct samples were fixed in formalin, processed and embedded in paraffin, and then sectioned with a microtome at 5-µm thickness, and stained with hematoxylin and eosin using a Varistain Gemini ES (Thermo Fisher).

### Immunofluorescent Staining for Detection of Hsc70

Cells were diluted in media, placed in cytospin funnels, and spun at 1,000 rpm for 8 min. The slides were fixed in 4% PFA solution for 15 min and washed twice in PBS for 5 min. They were next blocked with 5% normal donkey serum in 1% BSA in PBS for 1 h at room temperature. After being blocked, rat anti-Hsc70 primary antibody was applied at a 1:200 dilution in 1% BSA in PBS and incubated overnight at 4°C. The slides were washed three times in PBS for 10 min. Alexa Fluor 594-conjugated AffiniPure donkey anti-rat secondary antibody (Ref. 712-585-153, Jackson ImmunoResearch, West Grove, PA) was applied at a 1:250 dilution in 1% BSA in PBS and incubated for 1.5 h at room temperature. The slides were then washed 5 times in PBS, and coverslips were applied using VECTASHIELD Antifade Mounting Medium with DAPI (Vector Laboratories, Burlingame, CA).

### Immunohistochemistry for Detection of Natural Killer Cells in the Liver

Fixed liver slides were incubated at 65°C for 30 min and then placed in xylene for a total of 45 min. They were next rehydrated through a series of ethanol washes. The slides underwent retrieval in a 1:20 Dako Target Retrieval Solution (Aligent, Santa Clara, CA) and were placed in a pressure cooker for 30 min. Slides were washed with running water for 5 min followed by a 5-min PBS wash. These slides were next incubated for 15 min at room temperature in a 3% peroxide solution (Sigma-Aldrich) followed by two 5-min PBS washes. Liver section slides were blocked in a 1:10 solution of normal goat serum (Vector Laboratories, Burlingame, CA) in 1% BSA in PBS followed by a 10-min incubation with a milk-blocking solution. They were then incubated overnight at 4°C with rabbit anti-CD49b antibody (Ref. AB133557, Abcam) at a 1:100 dilution in 1% BSA in PBS.

The next day, slides were rinsed in water followed by three washes of PBS. Goat anti-rabbit secondary antibody (Ref. BA-1000, Vector Laboratories) was added at a 1:1,000 dilution and incubated at 4°C overnight. Slides were washed three times with PBS for 15 min per wash. Vectastain Elite ABC R.T.U. solution (Vector Laboratories) was added, and the slides were incubated at room temperature for 50 min. The slides were next washed with PBS 3 times for 10 min per wash. Slides were then placed in a DAB solution (Vector Laboratories), made of 80 drops of buffer, 118 drops of H_2_O_2_, and 150 drops of DAB in 200 mL of water, for 30 s. The slides were washed with running water for 5 min, washed three times in PBS for 15 min each, then counterstained in hematoxylin and washed with running water for 5 min. They were incubated in 0.5% ammonium hydroxide for 10 min, washed with running water for 5 min, and dehydrated through ethanol series. Coverslips were applied using Vectashield mounting media (Vector Laboratories).

### Detection of CXCL9 and CXCL10 by ELISA

Quantitative assessments of CXCL9 and CXCL10 levels in serum from 7 and 10 days postinfected WT-BALB/c and TLR3 KO pups were performed using an ELISA (R&D Systems, Minneapolis, MN). Each sample consisted of the combined serum from two pups. These samples were diluted 1:10 for CXCL9, and 1:15 for CXCL10 followed by assays performed as per the manufacturer’s instructions. The optical density was obtained using a Biotek Synergy H1 microplate reader (RRID:SCR_019748, Agilent, Santa Clara, CA) set to 450 nm and concentrations were calculated with software (RRID:SCR_017317, Biotek Gen5 v3.04, Agilent).

### Human Study Samples

The details of human subjects, serum samples, and clinical and histological data were previously reported (38).

### Statistical Analysis

Continuous variables were expressed as means ± SE and were evaluated by ANOVA with Tukey’s post hoc testing, where appropriate. Analysis of noncontinuous variables was performed by χ^2^ and Fisher exact testing. A *P* value < 0.05 was considered significant. All statistical analyses were completed using Prism 9.5.0 (GraphPad, RRID:SCR_002798, Software, Inc., La Jolla, CA).

## RESULTS

### Rotavirus Method of Entry

**Figure 1.**
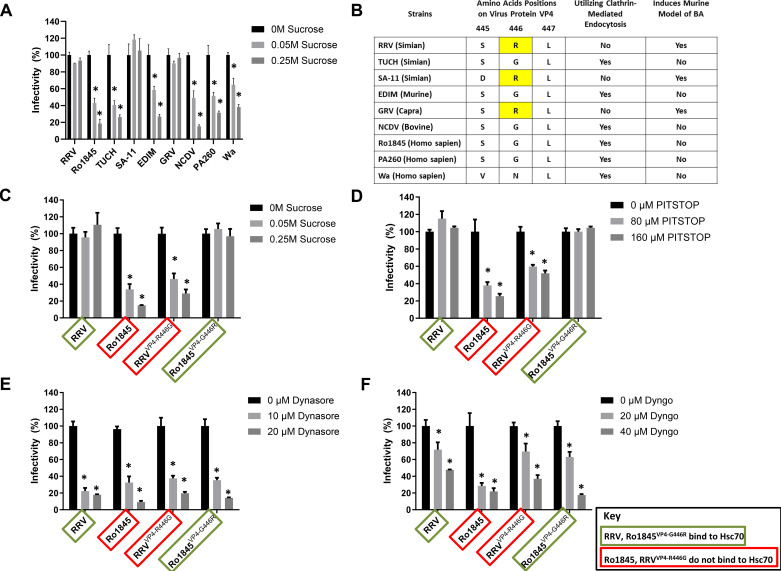
Effect of inhibition of clathrin-mediated and dynamin-mediated endocytosis on viral replication. *A*: after sucrose treatment, viral titers significantly decreased in TUCH, EDIM, NCDV, Ro1845, PA260, and Wa infections, whereas RRV, SA-11, and GRV titers were not affected. *B*: the viral titer of those strains containing the amino acid sequence arginine (R) at amino acid position 446 on their VP4 protein was not affected following sucrose treatment, to block clathrin-mediated endocytosis. *C*: sucrose treatment of cholangiocytes significantly decreased the viral titers of both strains unable to bind to Hsc70, Ro1845, and RRV^VP4-R446G^, while Hsc70-binding strains RRV and Ro1845^VP4-G446R^ showed no change in viral titers. *D*: treatment of cholangiocytes with the chemical inhibitor PITSTOP revealed a decrease in Ro1845 and RRV^VP4-R446G^ titers, while the viral titers of RRV and Ro1845^VP4-G446R^ saw no decrease. *E*: treatment with Dynasore, an inhibitor of dynamin-mediated endocytosis before infection revealed a decrease in titers of all viruses tested. *F*: cholangiocyte treatment with Dyngo showed a decrease in titers following infection with all viruses. For all experiments, values (*n* = 3 replicates) are expressed as percentage of control with standard errors; each assay was repeated three times.*P < 0.05. Hsc70, heat shock cognate 70 protein; RRV, rhesus rotavirus.

To determine the mechanism of entry after binding, we treated cholangiocytes with sucrose to remove clathrin-coated pits followed by infection with multiple strains of rotavirus from several species. Sucrose treatment significantly decreased the viral titers of TUCH, EDIM, NCDV, Ro1845, PA260, and Wa, while RRV, SA-11 and GRV demonstrated no significant difference (Fig. 1*A*). Analysis of the VP4 protein amino acid sequences of these strains revealed all strains unaffected by the removal of clathrin-coated pits for entry contained an arginine (R) at amino acid 446 (Fig. 1*B*). Previously, we demonstrated that RRV uses this amino acid as part of the sequence “SRL” at position 445–447 to bind to the Hsc70 protein on the surface of cells (10).

To determine if clathrin-mediated endocytosis of rotavirus is contingent on Hsc70 binding, we repeated this experiment using RRV, and Ro1845, along with RRV^VP4-R446G^, an RRV strain with a single mutation of arginine to glycine at amino acid 446 on the VP4 protein rendering it unable to bind to Hsc70, and Ro1845^VP4-G446R^, a Ro1845 strain containing a glycine to arginine mutation at amino acid 446 in the VP4 protein permitting binding to Hsc70. Sucrose treatment significantly reduced the viral titers of Ro1845 while also affecting RRV^VP4-R446G^, in contrast, RRV and Ro1845^VP4-G446R^ demonstrated no significant difference (Fig. 1*C*). To further validate these findings, we treated cholangiocytes with PITSTOP, a clathrin inhibitor, followed by infection with the same viruses. As before, Ro1845 and RRV^VP4-R446G^ displayed a significant decrease in viral titer while RRV and Ro1845^VP4-G446R^ titers remained unaffected (Fig. 1*D*).

Cholangiocytes were treated with Dynasore to affect dynamin-mediated endocytosis following infection. All viruses evaluated exhibited a significant decrease in viral titers (Fig. 1*E*). To validate this, we treated cholangiocytes with Dyngo, another dynamin inhibitor, and observed a similar decrease in viral replication of all viruses tested (Fig. 1*F*). These findings illustrate that the ability to bind to Hsc70 allows RRV to use a clathrin-independent, dynamin-dependent method for endocytosis.

### Viral Trafficking to Early and Late Endosomes

**Figure 2.**
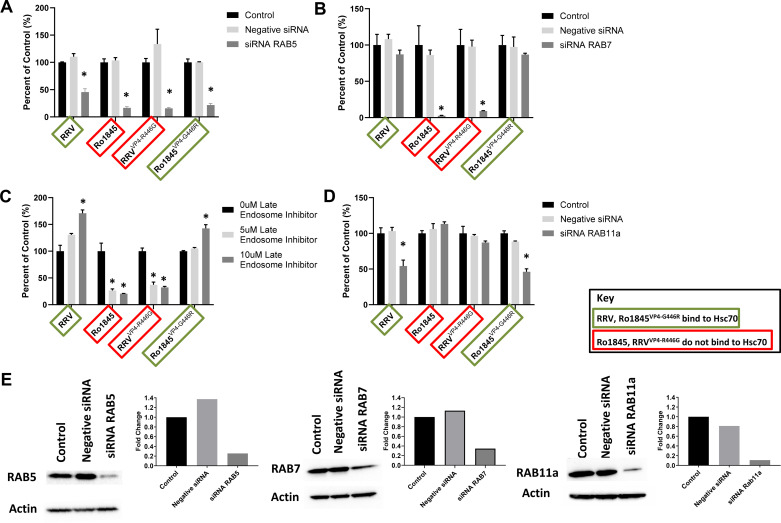
Rotavirus trafficking to early and late endosomes. *A*: cholangiocytes treated with siRNA against RAB5, a protein associated with the early endosome, followed by infection with either RRV, Ro1845, RRV^VP4-R446G^, or Ro1845^VP4-G446R^ showed a decrease in all the viruses. *B*: treatment of cholangiocytes with siRNA against RAB7, a protein associated with the late endosome, revealed decreased titers for Ro1845 and RRV^VP4-R446G^ but no change in titers of RRV and Ro1845^VP4-G446R^. *C*: cholangiocyte treatment with late endosome inhibitor demonstrated a significant decrease in the viral titers of Ro1845 and RRV^VP4-R446G^ but a significant increase in the titers of RRV and Ro1845^VP4-G446R^. *D*: treatment of cholangiocytes with siRNA against RAB11a, a protein associated with formation of the recycling endosome, followed by infection with the viruses displayed a decrease in the titers of RRV and Ro1845^VP4-G446R^, while having no effect on viral titers of Ro1845 and RRV^VP4-R446G^. For all experiments, values (*n* = 3 replicates) are expressed as percentage of control with standard errors; each assay was repeated three times. *E*: Western blot analysis revealed that the targeted genes are appropriately silenced following treatment. siRNA treatment against RAB5 resulted in a 74% reduction of RAB5 signaling. RAB7 signaling showed a 66% reduction following siRNA treatment. Cholangiocytes treated with siRNA against RAB11a experienced an 89% reduction in RAB11a signaling. *P < 0.05. RRV, rhesus rotavirus.

To determine if this altered method of endocytosis affects endosomal trafficking, we treated cholangiocytes with siRNA against RAB5, a protein associated with the early endosome, and then infected cholangiocytes with the aforementioned rotavirus strains (39). All strains investigated displayed a decrease in infectivity (Fig. 2*A*).

We then treated cholangiocytes with siRNA against RAB7 to inhibit late endosome development followed by viral infection (39). Only Ro1845 and RRV^VP4-R446G^ showed a decrease in viral titer while RRV and Ro1845^VP4-G446R^ exhibited no change (Fig. 2*B*). Cholangiocytes treated with a Late Endosome Inhibitor demonstrated similar results with a significant decrease in the viral replication of Ro1845 and RRV^VP4-R446G^, while demonstrating an increase in RRV’s and Ro1845^VP4-G446R^’s viral titers (Fig. 2*C*).

Furthermore, we inhibited the formation of the recycling endosome using siRNA against RAB11a and then infected cholangiocytes with our viruses. Inhibition led to a decrease in viral titer of RRV and Ro1845^VP4-G446R^, while having no effect on Ro1845 and RRV^VP4-R446G^ (Fig. 2*D*). Western blots revealed all siRNA treatments appropriately reduced signaling of targeted genes (Fig. 2*E*). These data suggest rotaviruses that use Hsc70 do not traffic to the late endosome, but instead traffic from the early endosome to the recycling endosome where the virus proceeds to uncoat.

### “SRL” Motif Binding Site on Hsc70

**Figure 3.**
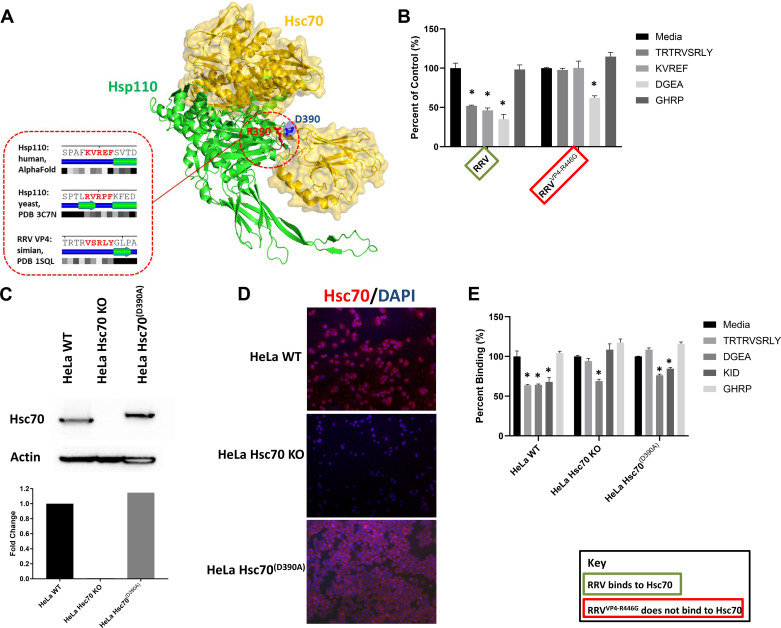
The involvement of D390 in RRV infection. *A*: the X-ray-resolved crystal structure of Hsp110 *S. cerevisiae* protein in complex with Hsc70 (*right*), with the critical pair of interacting residues, R390 of Hsp110 and D390 of Hsc70, shown in red and blue, respectively. The sequences and local structures of the R390 containing loops in Hsp110 orthologs in human (predicted using AlphaFold) and yeast (experimentally resolved) are shown in the *left* and aligned with their predicted counterpart in RRV VP4 protein in the *bottom*. The RRV VP4 structural counterpart contains the “SRL” motif within a similar local environment as defined by sequence, secondary structure, and solvent accessibility similarity. The amino acid sequence is shown in the top row with red letters indicating the interaction site. Observed or predicted secondary structures are shown in the middle row, with blue lines indicating loops and green fragments corresponding to beta strands, while solvent accessibilities are shown in the bottom rows with black, dark, and white boxes corresponding to buried, partially buried, and exposed residues, respectively. *B*: treatment of cholangiocytes with the synthetic peptides TRTRVSRLY, KVREF, DGEA, and GHRP demonstrated a decrease in RRV infectivity following TRTRVSRLY, KVREF, and DGEA treatment but in contrast infection of RRV^VP4-R446G^ was only inhibited by DGEA. *C*: Western blot demonstrated that the Hsc70 protein was not detectable in HeLa Hsc70 KO but was present in the HeLa WT and HeLa Hsc70^(D390A)^. *D*: immunofluorescent staining for Hsc70 also illustrated that Hsc70 was not detectable on the HeLa Hsc70 KO cells while it was present on the surface of HeLa WT and HeLa Hsc70^(D390A)^ cells. *E*: HeLa WT, HeLa Hsc70 KO, and HeLa Hsc70^(D390A)^ were treated with the peptides TRTRVSRLY, DGEA, KID, and GHRP preceding RRV infection. HeLa WT cells experienced decreased infectivity of RRV following treatment with TRTRVSRLY, DGEA, and KID. HeLa Hsc70 KO only experienced a decrease in RRV infectivity after DGEA treatment. HeLa Hsc70^(D390A)^ saw decreased RRV infectivity after DGEA and KID treatment. For all experiments, values (*n* = 3 replicates) are expressed as percentage of control with standard errors; each assay was repeated three times. *P < 0.05. Hsc70, heat shock cognate 70 protein; RRV, rhesus rotavirus.

To identify where RRV is binding on Hsc70, we analyzed native Hsc70 complexes, including a protein-protein interaction between Hsp110 and Hsc70 identified by Shuermann et al. (40). A structurally resolved complex of Hsc70 and Hsp110 indicates that the Hsp110 linker fragment SPAFKVREF mediates the interaction with Hsc70, with R390 contributing most to binding energy via interaction with D390 on Hsc70. This sequence is similar to the RRV “SRL” site and aligns as follows: SPAFKVREF/TRTRVSRLY. We hypothesize that RRV’s VP4 uses “SRL” to bind to Hsc70 at the same binding site as Hsp110. On Hsc70, the interacting counterpart involves linker residues 390–400: DLLLLDVTPLS, where D390 makes direct contact with R390 of the Hsp110 linker (Fig. 3*A*).

To confirm that D390 on Hsc70 is essential for RRV binding, we pretreated cholangiocytes with the synthetic peptides TRTRVSRLY (against “SRL” binding site on Hsc70), KVREF (against Hsp110 linker fragment), DGEA (against integrin α2β1), or GHRP (negative control) followed by viral infection with RRV or RRV^VP4-R446G^. Peptides TRTRVSRLY, KVREF, and DGEA all resulted in a significant decrease in viral titers of RRV while titers for RRV^VP4-R446G^ only decreased following DGEA treatment (Fig. 3*B*), showing that D390 is integral in allowing RRV to bind to Hsc70.

### The Role of Amino Acid D390 on Hsc70 in Viral Entry and Trafficking

To validate the D390 binding site on Hsc70, we transduced HeLa Hsc70 KO using a lentivirus containing a mutant form of Hsc70, with aspartic acid at amino acid 390 altered to an alanine, creating a HeLa line with a mutated Hsc70 [HeLa Hsc70^(D390A)^], which was illustrated by Western blot and immunocytochemistry (Fig. 3, *C* and *D*). To confirm the presence of RRV VP4’s binding sites, each cell type was pretreated before RRV infection with synthetic peptides TRTRVSRLY, DGEA, GHRP, and KTKIDRSTQISPNTLPD (KID) (against amino acid 642–658 on Hsc70), which has previously been shown to block secondary attachment by all rotavirus strains to Hsc70 (10, 41). HeLa WT cells exhibited a decrease in viral titers after treatment with TRTRVSRLY, DGEA, and KID similar to previously published experiments performed on cholangiocytes (10). HeLa Hsc70 KO cells demonstrated a decrease in RRV infectivity only after DGEA treatment while HeLa Hsc70^(D390A)^ displayed a decrease in titers following DGEA and KID treatment while in contrast, TRTRVSRLY had no effect (Fig. 3*E*). These data show that only the target amino acid was altered in the HeLa Hsc70^(D390A)^ cells, as RRV was still able to infect DGEA- and KID-treated cells.

**Figure 4.**
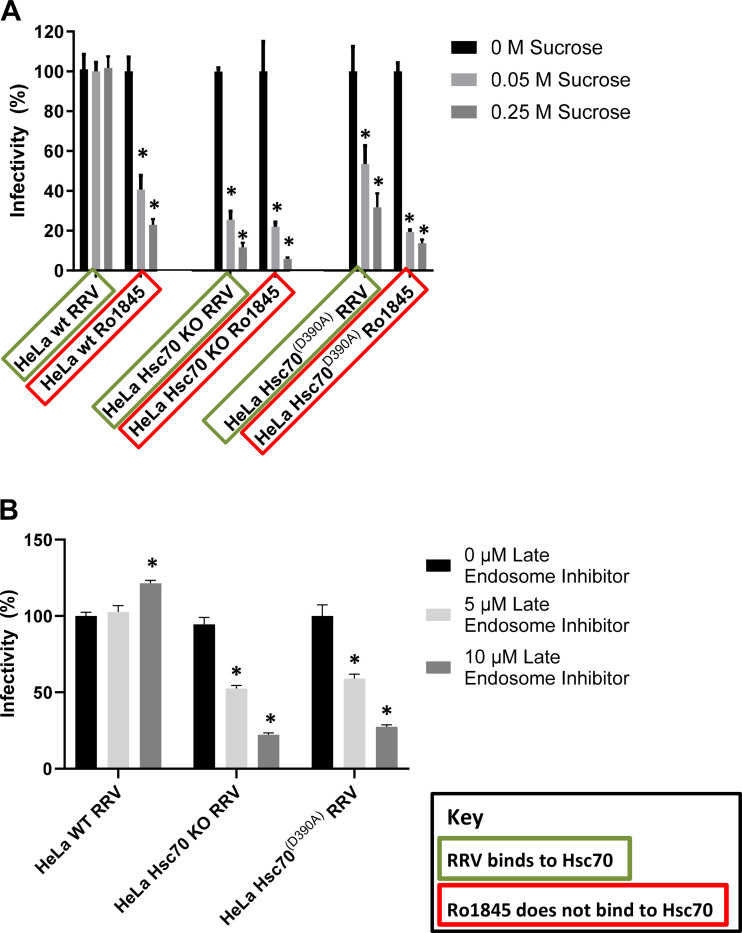
Inhibition of early and late endosome trafficking in WT and mutant HeLa cells. *A*: treatment of HeLa WT, HeLa Hsc70 KO, and HeLa Hsc70^(D390A)^ cells with sucrose resulted in a decrease in infectivity of RRV and Ro1845 in all cell types except the infectivity of RRV in HeLa WT, which experienced no change. *B*: inhibiting late endosome trafficking in these cells using a late endosome inhibitor resulted in decreased RRV titers in infected HeLa Hsc70 KO and HeLa Hsc70^(D390A)^ cells and increased titers in infected HeLa WT cells. For both experiments, values (*n* = 3 replicates) are expressed as percentage of control with standard errors; each assay was repeated three times.*P < 0.05. Hsc70, heat shock cognate 70 protein; RRV, rhesus rotavirus.

To determine if loss of Hsc70 alters the endocytic pathway used by RRV, the three HeLa cell lines were treated with sucrose to inhibit clathrin-mediated endocytosis followed by infection with either RRV or Ro1845. In HeLa WT cells, only the Ro1845-infected cells revealed a decrease in infectivity while having no effect on the ability of RRV to infect, paralleling the cholangiocytes. Notably, infectivity was significantly reduced for both RRV and Ro1845 following sucrose treatment in HeLa Hsc70 KO and HeLa Hsc70^(D390A)^ cell lines (Fig. 4*A*). To determine whether altering the method of endocytosis affected intracellular viral trafficking, cells were treated with the Late Endosome Inhibitor. RRV-infected HeLa WT cells experienced an increase in viral titers following treatment, as seen previously in cholangiocytes, demonstrating replication does not require trafficking to the late endosome. Conversely, infection of both the HeLa Hsc70 KO and HeLa Hsc70^(D390A)^ cell lines revealed a significant decrease in RRV viral titers indicating that late endosome trafficking is necessary for replication (Fig. 4*B*). These data suggest that “SRL” binding to Hsc70 governs RRV’s method of entry and trafficking within the cell.

### Activation of NF-κB Signaling Pathway in Cholangiocytes and Cytokine Release from Cholangiocytes following RRV Infection

**Figure 5.**
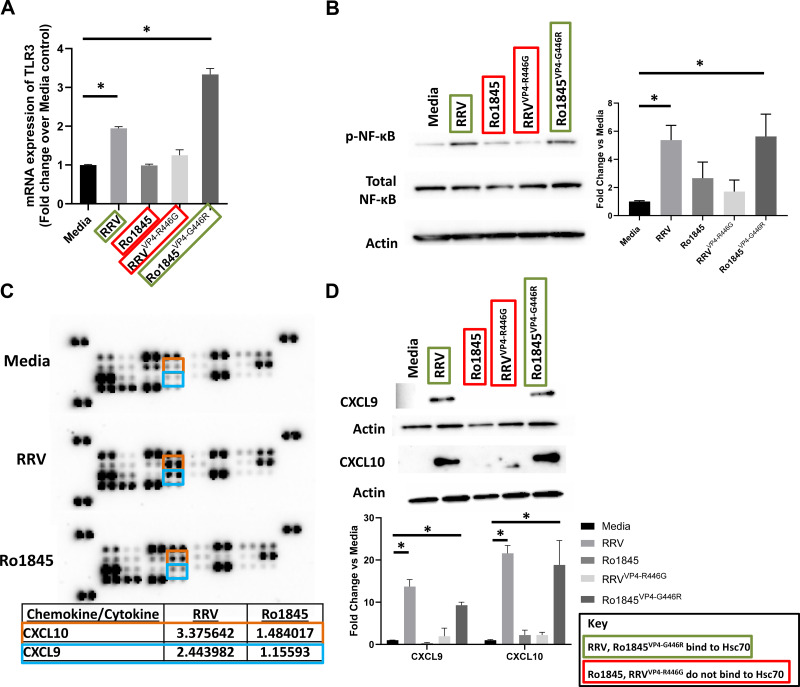
Activation of the TLR3-NF-κB pathway and subsequent cytokine release. *A*: real-time PCR performed on cholangiocytes 8 h postinfection with RRV and Ro1845^VP4-G446R^ demonstrated significantly higher levels of *TLR3* expression than Ro1845 and RRV^VP4-R446G^ infections. *B*: Western blot analysis of lysate from cholangiocytes infected with RRV, Ro1845, RRV^VP4-R446G^, or Ro1845^VP4-G446R^ revealed higher levels of phosphorylated NF-κB after RRV and Ro1845^VP4-G446R^ infection than Ro1845 and RRV^VP4-R446G^ infection. *C*: protein array specific for chemokines displayed high levels of CXCL9 and CXCL10, outlined in blue and orange respectively, following RRV infection while this increase was not seen following Ro1845 infection. Fold change was reported versus media-only treatment. *D*: Western blot analysis revealed significantly high levels of CXCL9 and CXCL10 in cholangiocyte supernatant following RRV and Ro1845^VP4-G446R^ infection, which was undetected following Ro1845 and RRV^VP4-R446G^ infection. Real-time PCR (*n* = 3 replicates) is expressed as a fold change over media treatment and Western blot values (*n* = 3 replicates) are expressed as mean densitometry with standard error; both assays were repeated three times. *P < 0.05. RRV, rhesus rotavirus.

After demonstrating that RRV traffics to the early recycling endosome following cell entry, we determined if it activates downstream pathways. mRNA expression of *TLR3* from cholangiocytes revealed a significantly higher expression in RRV- and Ro1845^VP4-G446R^-infected cholangiocytes than those infected with Ro1845 and RRV^VP4-R446G^ (Fig. 5*A*).

Because NF-κB is known to be downstream of TLR3, we next analyzed if our viruses could stimulate its phosphorylation. Western blot analysis of the cell lysate of infected cholangiocytes after infection with either RRV, Ro1845, RRV^VP4-R446G^, or Ro1845^VP4-G446R^ revealed phosphorylated NF-κB was significantly increased in RRV- and Ro1845^VP4-G446R^-infected cholangiocytes than Ro1845 and RRV^VP4-R446G^ infection (Fig. 5*B*). A protein array used to detect key cytokines in the supernatant of RRV- and Ro1845-infected cholangiocytes revealed CXCL9 and CXCL10 to be the most prevalent cytokines released following RRV infection after normalization to mock-infected supernatant (Fig. 5*C*). Confirmatory Western blots performed on RRV- and Ro1845^VP4-G446R^-infected cell culture supernatants revealed increased levels of cytokines CXCL9 and CXCL10 while Ro1845 and RRV^VP4-R446G^ infection did not (Fig. 5*D*).

**Figure 6.**
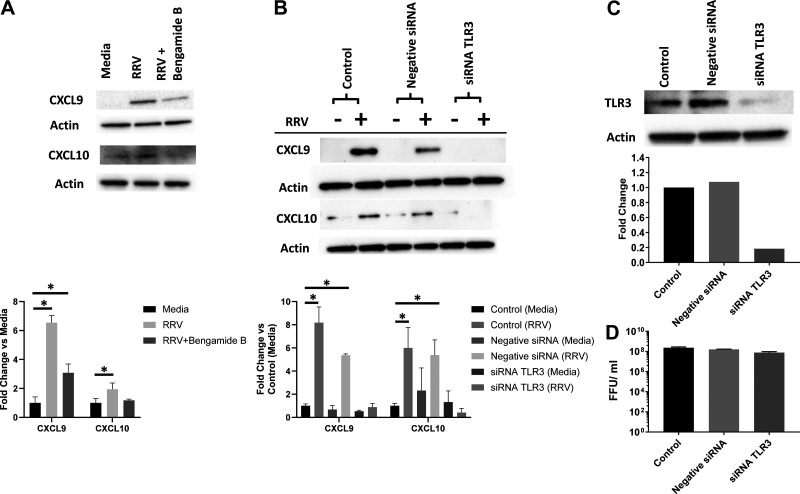
Effect of TLR-3 and NF-κB inhibition on cytokine release. *A*: Western blot analysis showed that supernatant from cholangiocytes treated with Bengamide B to inhibit NF-κB release had significantly lower levels of CXCL9 and CXCL10 release following RRV infection compared with untreated cells. *B*: inhibiting TLR3 activation by treating cholangiocytes with siRNA against TLR3 resulted in a decrease in CXCL9 and CXCL10 following RRV infection, while no decrease was seen in infected cholangiocytes without siRNA treatment. Western blot values (*n* = 3 replicates) are expressed as mean densitometry with standard error; the assay was repeated three times. *C*: siRNA treatment against *TLR3* led to an 82% reduction in TLR3 signaling. *D*: cholangiocytes treated with siRNA against *TLR3* revealed no decrease in viral replication (*n* = 3 replicates/group). *P < 0.05. RRV, rhesus rotavirus; TLR3, Toll-like receptor 3.

We next investigated whether release of these cytokines was driven by NF-κB and TLR3 signaling. We treated cholangiocytes with Bengamide B, an NF-κB pathway inhibitor, which significantly inhibited the release of both CXCL9 and CXCL10 as revealed by Western blot (Fig. 6*A*). Next, cholangiocytes treated with siRNA against TLR3 resulted in a significant decrease in both CXCL9 and CXCL10 in RRV-infected cholangiocytes after treatment suggesting that TLR3 activation led to their release (Fig. 6*B*). This treatment both effectively knocked down TLR3 signaling and did not inhibit RRV’s ability to replicate (Fig. 6, *C* and *D*). The increase in NF-κB signaling and cytokine release following RRV infection demonstrates a possible pathway that is being activated through the RRV infection pathway initiated by Hsc70 binding.

### Serum Levels of CXCL9 and CXCL10 in Infected Pups

**Figure 7.**
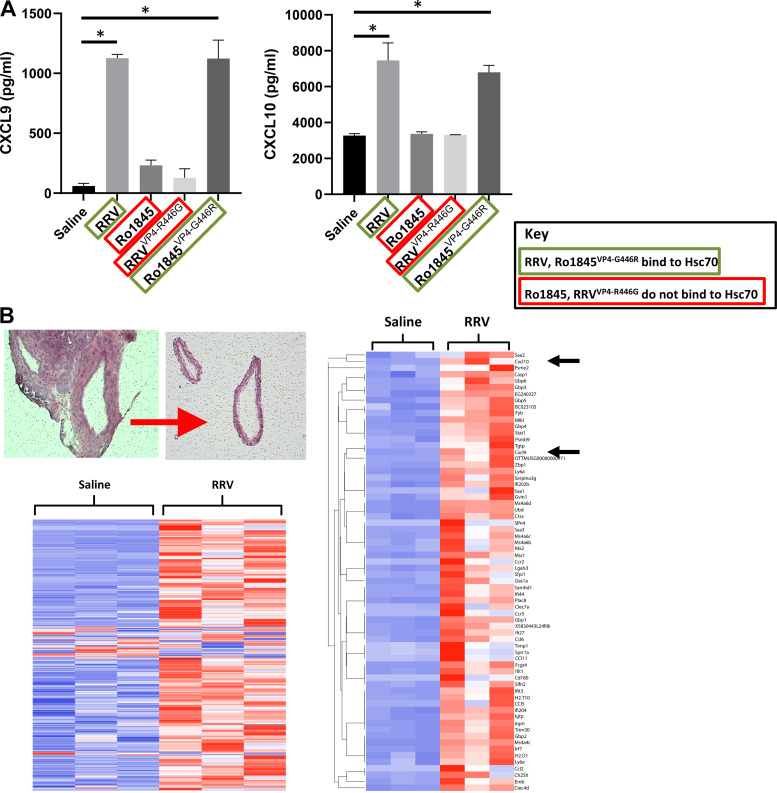
Cytokine detection in vitro and in vivo. *A*: serum evaluated from 7 days post-RRV, Ro1845-, RRV^VP4-R446G^-, or Ro1845^VP4-G446R^ (*n* = 3 for each group)-infected pups demonstrated significantly higher levels of CXCL9 and CXCL10 in RRV and Ro1845^VP4-G446R^ than those mice infected with Ro1845 or RRV^VP4-R446G^ as determined by ELISA. *B*: microarray analysis performed on biliary epithelial cells isolated using LCM from livers of 7 days post-RRV-infected mice expressed a greater than fivefold increase in CXCL9 and CXCL10 as depicted in the subset heatmap. *P < 0.05. RRV, rhesus rotavirus.

With the understanding that RRV and Ro1845^VP4-G446R^ can induce the murine model of biliary obstruction, we wanted to evaluate CXCL9 and CXCL10 levels in vivo following infection. ELISAs for CXCL9 and CXCL10 were performed on serum harvested from pups 7 days postinfected revealed an increase in both CXCL9 and CXCL10 in mice infected with RRV and Ro1845^VP4-G446R^, while there was no significant difference after Ro1845 or RRV^VP4-R446G^ infection (Fig. 7*A*). To determine if these cytokines were released by infected cholangiocytes, we performed cDNA microarray analysis on mRNA isolated from the extrahepatic bile duct of mice injected with RRV or normal saline using LCM and demonstrated that CXCL9 and CXCL10 are both highly expressed in infected biliary epithelial cells (Fig. 7*B*).

### RRV Infection in TLR3 KO Mice

**Figure 8.**
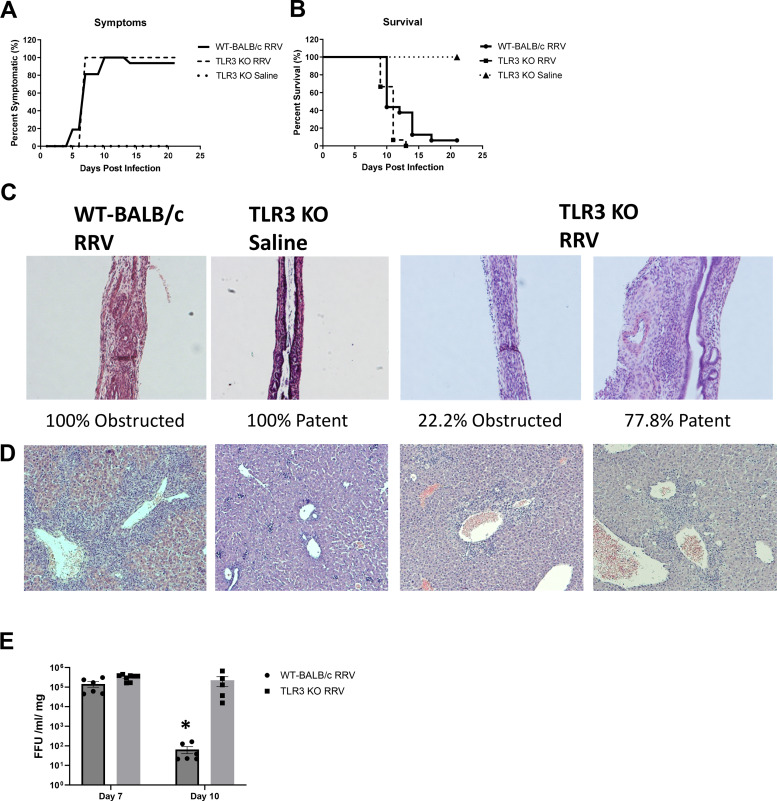
RRV infection in TLR3 KO mice. *A*: TLR3 KO mice and WT-BALB/c mice infected with RRV experienced 100% symptomatology with 100% mortality (*B*) of TLR3 KO mice and 93% of WT-BALB/c by Kaplan-Meier survival analysis. *C*: hematoxylin and eosin staining revealed 100% of WT-BALB/c-infected mice had an obstructed bile duct while in contrast only 22.2% of RRV-infected TLR3 KO were obstructed (*n* = 4 pups) leaving 77.8% (*n* = 14 pups) with patent bile ducts. *D*: hematoxylin and eosin-stained liver section displayed an increase in inflammation following RRV infection regardless of mice genotype or bile duct patency. *E*: at *day 10*, viral titers were significantly higher in the bile ducts of TLR3 KO-infected mice when compared with WT-BALB/c-infected mice. *P < 0.05. RRV, rhesus rotavirus; TRL3, Toll-like receptor 3.

After discovering the role of TLR3 in CXCL9 and CXCL10 release, we sought to ascertain if TLR3 KO mice would attenuate the murine model of BA following RRV infection. All RRV-infected TLR3 KO mice developed obstructive symptoms and experienced the same rate of mortality as infected WT-BALB/c mice (Fig. 8, *A* and *B*). Interestingly, only 22.2% of infected TLR3 KO pups developed extrahepatic bile duct obstructions while the remaining 77.8% remained patent (Fig. 8*C*). However, even with this patency, the liver sections still displayed increased levels of inflammation similar to the obstructed TLR3 KO and WT-BALB/c mice (Fig. 8*D*). Viral titers performed on bile ducts harvested from WT-BALB/c and TLR3 KO mice 7 and 10 days post-RRV infection demonstrated equal infection at *day 7* while *day 10* post-RRV-infected TLR3 KO mice have significantly higher viral titers than WT-BALB/c mice, indicating an ongoing, unregulated viremia (Fig. 8*E*).

**Figure 9.**
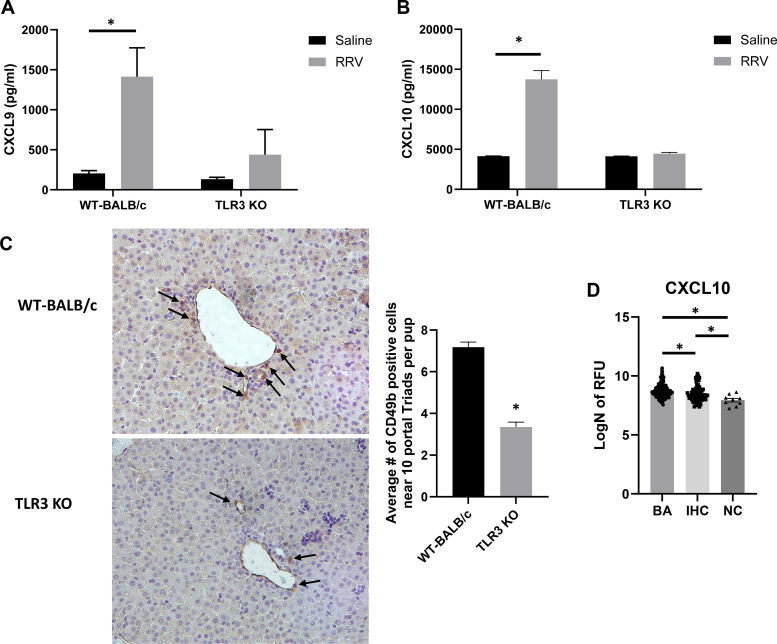
Cytokine levels in TLR3 KO mice and analysis of human patient samples. *A*: CXCL9 levels were significantly higher in RRV-infected WT-BALB/c mice (*n* = 3 pups) while no significant difference was observed in RRV-infected TLR3 KO mice (*n* = 3 pups) compared with saline controls at 10 days postinfection. *B*: similarly, RRV-infected WT-BALB/c mice (*n* = 3 pups) experienced significantly higher levels of CXCL10 compared with saline control, while TLR3 KO-infected mice (*n* = 3 pups) did not. *C*: immunohistochemistry on RRV-infected TLR3 KO mice (*n* = 7 pups) revealed significantly fewer numbers of CD49b-positive cells (arrows) in the livers around portal triads (10 portal triads/pup) than WT-BALB/c mice (*n* = 4 pups). *D*: CXCL10 levels were significantly elevated in patients with BA compared with patients with IHC and normal controls as reported in SOMAscan data. *P < 0.05. BA, biliary atresia; IHC, intrahepatic cholestasis; RRV, rhesus rotavirus; TLR3, Toll-like receptor 3.

ELISA conducted on serum collected 10 days postinfection from both saline and RRV-infected WT-BALB/c and TLR3 KO mice revealed that WT-BALB/c mice expressed significantly higher levels of CXCL9 and CXCL10 after RRV infection while TLR3 KO mice show no significant increase in CXCL9 or CXCL10 following infection (Fig. 9, *A* and *B*). As noted previously, proinflammatory cytokines can recruit NK cells. To determine if CXCL9 and CXCL10 were recruiting NK cells in the murine model of BA, we performed immunohistochemistry for CD49b, an NK cell marker, on WT-BALB/c and TLR3 KO pups infected with RRV. There were significantly fewer CD49b-positive cells surrounding the portal triads of TLR3 KO mice compared with WT-BALB/c mice following RRV infection (Fig. 9*C*). These data show TLR3 deficiency is associated with a decrease in CXCL9 and CXCL10 levels along with a reduced number of NK cells.

### CXCL10 Levels in Patients with BA

To ascertain if these data are reflected in patients with BA, we analyzed the data obtained from patient-based SOMAscan assay performed on samples obtained from patients with BA (38). Patients with BA have significantly higher levels of CXCL10 at the time of HPE procedure when compared with both a disease control, intrahepatic cholestasis (IHC) and normal controls (Fig. 9*D*). This cytokine increase reveals a link between what was previously noted in the murine model and what is seen in the human disease process, suggesting a possible cause for the biliary obstruction seen in human patients with BA.

## DISCUSSION

Biliary atresia is a disease of infancy resulting from an obstruction of the intra- and extrahepatic bile ducts. If intervention is not performed, the disease progresses to liver fibrosis, end-stage liver disease, and mortality. Although the precise etiology of BA is unknown, a common hypothesis for onset of disease is a viral insult, which is supported by the detection of various viruses, including rotavirus in explanted patient livers (4, 42, 43). It is important to note that presence of CMV is detected in a subset of patients, between 10% and 24% as reported by various studies (44, 45). These patients experience lower rates of jaundice clearance following Kasai compared with patients without virus detected in their livers (46). Our laboratory has focused on how RRV induces the murine model of BA and has identified a novel peptide sequence “SRL” found within the viral attachment protein, VP4. Importantly, the “SRL” peptide has been found in the attachment protein of other viruses detected in patients with BA including reovirus, HPV, EBV, and CMV supporting the hypothesis that viral insult may lead to BA in a subset of patients (23). We have previously demonstrated that altering a single amino acid in this sequence, arginine (R) at amino acid 446 to guanine (G), inhibits RRV’s induction of the murine model of BA and conversely mutating the G at amino acid 446 to an R allows for the induction of the model by Ro1845 (24). In this study, we have further defined how this peptide sequence may govern intracellular trafficking altering the cellular host response leading to obstruction.

Rotaviruses typically use a clathrin-dependent pathway to gain cell entry and traffic to the early endosome followed by trafficking to the late endosome; however, RRV is unique in that it uses a clathrin-independent, dynamin-dependent method of endocytosis and does not traffic to the late endosome but instead traffics to the early recycling endosome, the mechanism of which is still unknown (11, 47, 48). We hypothesized that RRV’s “SRL” interaction with Hsc70 affects the viral trafficking pathway as we have previously demonstrated the amino acid sequence “SRL” expressed on RRV’s VP4 protein governs attachment by binding to Hsc70 expressed on the cell surface and altering this sequence inhibits the strain’s ability to induce murine BA (10, 24). Two other rotavirus strains capable of inducing the murine model of BA, SA-11, and GRV also contain an arginine at amino acid 446 on their VP4 proteins (6, 10, 24). This observation strengthens the hypothesis that the peptide sequence “SRL” contributes to the unique cholangiocyte infection leading to murine BA.

To elucidate how binding to Hsc70 alters the viral pathway, we first focused on how the virus enters the cell. After inhibition of clathrin-mediated endocytosis in cholangiocytes, we infected these cells with either RRV, Ro1845^VP4-G446R^, which can bind to Hsc70 or Ro1845, RRV^VP4-R446G^, which cannot. We discovered inhibiting clathrin-mediated endocytosis decreased the titers of only those viruses that cannot bind to Hsc70. Martín et al. (49) demonstrated previously that RRV uses a dynamin-dependent, clathrin-independent pathway of endocytosis. To expand on these findings, we used Dyngo and Dynasore to inhibit the dynamin pathway and found a decrease in the titers of all viruses, confirming that an RRV and Ro1845^VP4-G446R^ viruses use a clathrin-independent, dynamin-dependent method of endocytosis.

After attachment, viruses enter cells and traffic through a particular pathway, usually moving from the early endosome to the late endosome where they uncoat and begin the replication process. Wolf et al. (48) has previously shown that RRV differs from this, stopping its trafficking in the early endosome without moving to the late endosome. When we inhibited early endosome trafficking, a part of trafficking required by all studied virus strains, with siRNA against RAB5, we saw a decrease in the infectivity of all of our viruses. Late endosome inhibition showed a decrease in the infectivity of our viruses that do not bind to Hsc70. When we inhibited the formation of the recycling endosome with siRNA against RAB11a, we saw a decrease in the infectivity of our Hsc70-binding viruses. With these data, we have defined the pathway used by viruses with the “SRL” peptide. This intracellular trafficking pathway may be the basis by which RRV causes the distinct immune response resulting in murine BA. Further research is needed to understand why RRV does not require trafficking to the late endosome.

After determining a mutation on RRV’s VP4 protein affects endocytosis, we sought to understand how WT-RRV would interact with a mutated Hsc70. Interestingly, when we alter a single amino acid on the binding site of Hsc70, aspartic acid at position 390 to alanine, on HeLa cells, we see RRV can no longer bind to Hsc70 and uses a clathrin-mediated method of endocytosis followed by trafficking to the late endosome. These data demonstrate that “SRL” binding to Hsc70 alters the method of endocytosis from clathrin-dependent to clathrin-independent and altering this receptor forces these viruses to use a clathrin-mediated method of endocytosis. This suggests it is not the default pathway of a specific virus, but rather a unique trafficking pathway initiated by Hsc70 binding.

We next sought to determine how this alternate endocytic and trafficking pathway results in the induction of the murine model of BA. Previous studies have shown that the TLR3-NF-κB pathway is activated following infection with dsRNA viruses leading to proinflammatory cytokine release (25, 29, 50). Interestingly, of the viruses studied here, we only observed activation of this pathway following infection with our early penetrating, Hsc70-binding viruses. These cytokines in turn recruit NK cells causing infected and noninfected cholangiocyte death leading to worse outcomes following RRV infection (31). Our studies revealed an increase in *TLR3* expression and phosphorylation of NF-κB following RRV and Ro1845^VP4-G446R^ infection, as well as an increase in the cytokines CXCL9 and CXCL10. Removal of MyD88, which signals through all TLRs with the exception of TLR3, is not protective against the murine model of BA, further suggesting the role of TLR3 in our BA model (51). When we inhibited NF-κB and TLR3 separately we did not see an increase in CXCL9 and CXCL10 even following infection with our Hsc70-binding viruses. These experiments provide data showing CXCL9 and CXCL10 release are contingent on TLR3 and NF-κB activation in cholangiocytes. Significant levels of these cytokines are released in vivo following RRV infection. The release of these cytokines following infection defines a mechanistic basis for the recruitment of NK cells to the liver, which we have previously shown leads to obstruction of the extrahepatic bile duct. Depletion of these NK cells is protective against biliary damage following infection as evidenced by Shivakumar et al. (31, 52).

Infection of TLR3 KO mice with RRV resulted in bile duct obstruction of only 22.2%, a sharp contrast to the 100% of RRV-infected WT-BALB/c mice with obstructed bile ducts, though they experienced similar symptomatology and mortality (34). Our results demonstrate that TLR3 deficiency alters the obstructive aspect of murine BA. More work is needed to define why TLR3 KO mice experience mortality without bile duct obstruction. We postulate their symptomatology and mortality may be due to ongoing liver inflammation and a massive viremia as these mice have significantly higher levels of viral titers at day 10 of life when compared with WT-BALB/c mice demonstrating they are unable to clear the virus. When we analyzed the serum of these RRV-infected TLR3 KO and WT-BALB/c mice, we found CXCL9 and CXCL10 was significantly reduced in the TLR3 KO mice and immunohistochemistry showed lower CD49b-positive (NK) cells.

Analysis of the data obtained from the SOMAscan assay performed on serum samples obtained from patients with BA enrolled in the ChiLDReN-sponsored PROBE trial reveal significantly higher levels of CXCL10 when compared with IHC and normal control groups (38). These data present a possible viral immune pathway leading to the recruitment of NK cells to the livers of BA-afflicted patients, leading to duct obstruction. Further research is needed to detail how the immune response in humans parallels that seen in mice.

**Figure 10.**
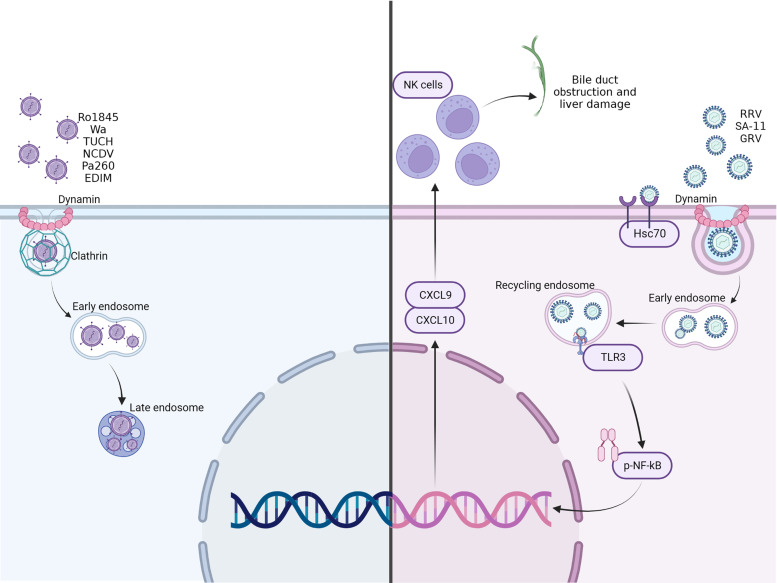
Graphical summary of viral entry process. Rotaviruses that contain amino acid “SRL” at amino acid 445-447 on VP4 bind to Hsc70 on the surface of cholangiocytes use a dynamin-mediated entry pathway trafficking to the early endosome but do not advance to the late endosome. In the early recycling endosome TLR3 and NF-κB become activated, leading to the release of proinflammatory cytokines CXCL9 and CXCL10. These cytokines attract NK cells to the site of inflammation where they cause cholangiocyte cell death, bile duct obstruction, and liver damage. Other rotavirus strains that do not bind to Hsc70 enter through a clathrin-mediated pathway, trafficking to the early endosome and eventually to the late endosome.

In summary, our data suggest that RRV binding to Hsc70 determines the endocytic pathway used to enter the cell, altering its intracellular trafficking, and initiating a possible mechanism leading to NK cell recruitment and subsequent bile duct obstruction (Fig. 10). Further studies focusing on these findings are required to develop potential therapies to alter the course of BA in human patients.

## DATA AVAILABILITY

Data will be made available upon reasonable request.

## SUPPLEMENTAL DATA

10.6084/m9.figshare.25848289 Supplemental Table S1: https://doi.org/10.6084/m9.figshare.25848289.

## GRANTS

This work was supported in part by National Institutes of Health Grants R01 DK-091566 (to G.T and S.K.M). J. M. gratefully acknowledges partial support by National Institutes of Health Grants R21AI097936, R01CA122346, P30ES006096, and U54HL127624.

## DISCLOSURES

No conflicts of interest, financial or otherwise, are declared by the authors.

## AUTHOR CONTRIBUTIONS

B.D., S.K.M., A.B., M.M., and G.T. conceived and designed research; H.T., B.D., S.K.M., S.M., H.M.P., S.H., A.B., and K.O. performed experiments; H.T., B.D., S.K.M., S.M., H.M.P., R.P., S.H., A.S., R.M., A.B., J.M., A.G.J., K.O., and G.T. analyzed data; A.B., B.D., S.K.M., R.P., R.M., J.M., A.G.J., M.M., P.S., and G.T. interpreted results of experiments; H.T., B.D., A.S., and J.M. prepared figures; H.T., H.M.P., R.P., S.H., A.S., A.B., J.M., and K.O. drafted manuscript; H.T., B.D., S.K.M., S.M., H.M.P., R.P., S.H., A.S., R.M., A.B., J.M., A.G.J., M.M., P.S., and G.T. edited and revised manuscript; H.T., B.D., S.K.M., S.M., H.M.P., R.P., S.H., A.S., R.M., A.B., J.M., A.G.J., M.M., P.S., and G.T. approved final version of manuscript.
